# Physiotherapy in German Palliative Care—A Nationwide Survey Among Physiotherapists

**DOI:** 10.3390/cancers17081311

**Published:** 2025-04-13

**Authors:** Paula Vradelis, Anna Elisabeth Pape, Martin Gschnell, Christian Volberg

**Affiliations:** 1 Department of Anaesthesiology & Intensive Care Medicine, Faculty of Medicine, Philipps University of Marburg, 35043 Marburg, Germany; 2 Department of Therapy Somatic, Klinikum Bremen-Ost, 28325 Bremen, Germany; 3 Department of Dermatology and Allergology, Faculty of Medicine, Philipps University of Marburg, 35043 Marburg, Germany; 4 Research Group Medical Ethics, Faculty of Medicine, Philipps University of Marburg, 35043 Marburg, Germany

**Keywords:** physiotherapy, palliative care, supportive therapy, end-of-life care

## Abstract

Objective: The aim of physiotherapy interventions for palliative care patients is to relieve pain, promote active movement and thereby improve quality of life and wellbeing. Physiotherapy is an important adjunct to medical and pharmacological treatments. The aim of this study is to identify the specific challenges, self-perceptions and therapeutic approaches of physiotherapists working in palliative care. Methods: An online survey of German physiotherapists was conducted using a 22-question questionnaire. The quantitative data were analysed descriptively (frequencies and percentages), and the qualitative responses were reviewed and grouped thematically. Results: A total of 450 valid responses were evaluated, of which 349 respondents worked in palliative care. The most common treatments for palliative care patients are respiratory therapy (85.8%), general physiotherapy measures (82.4%) and massages (72.9%). The analysis of the free-text responses identified five topics representing the work and challenges of physiotherapists in palliative care. A key issue identified is that the standard 20 min therapy session covered by health insurance is often insufficient to meet the individual needs and daily conditions of each patient. Physiotherapists with no previous experience of palliative care patients often require additional training and often feel inadequately prepared to work effectively with this patient population without appropriate further training. Many physiotherapists believe that they are involved in treatment too late. In addition, other healthcare professionals may not fully recognise the scope of their expertise. Conclusions: Physiotherapy in palliative care requires sensitivity, adaptability and sufficient time. Further training and early involvement are necessary to better address patients’ needs and harness the therapeutic potential of physiotherapists.

## Simple Summary

This research paper presents the findings of a nationwide survey among physiotherapists in Germany, focusing on their roles, experiences and challenges in palliative care settings. This study highlights the tasks of physiotherapists in palliative care and identifies the need for targeted training. By providing a comprehensive overview of current practices and research needs in palliative physiotherapy, this study aims to enhance the understanding of their essential role and promote interdisciplinary collaboration. The findings are intended to help improve patient-centred care strategies in palliative settings.

## 1. Introduction

Palliative care focuses on providing comprehensive support to people in the final phase of their lives, addressing both medical and psychosocial challenges. Patients receiving palliative care often experience a range of conditions affecting physical, psychological, social and spiritual aspects, such as pain, breathing difficulties and emotional distress [1,2]. Addressing these multiple challenges requires the collaboration of an interdisciplinary and multi-professional team, in which physiotherapy plays a crucial role alongside medical and nursing care [3,4].

Physiotherapy is seen as a valuable addition to the treatment options available, both by physiotherapists and by other healthcare professionals [5]. Physiotherapists address the physical and emotional needs of palliative patients through a comprehensive treatment approach, helping seriously ill patients to maintain the highest possible quality of life in their difficult circumstances. Traditional physiotherapy methods are typically used to maintain, restore or improve muscle strength, joint function and mobility. However, these aspects are not the primary focus in the treatment of palliative care. Instead, therapists must consider the needs of critically ill patients, whose conditions are often changing rapidly [6]. For example, the focus is on maintaining or improving remaining physical abilities to support patient independence. At the same time, symptoms such as pain, dyspnoea, loss of appetite, fatigue, lack of energy and oedema can be alleviated through physiotherapeutic interventions, including massage to relax muscles, manual lymphatic drainage, cold and heat applications and respiratory therapy, providing relief throughout the end of life [7].

Wünsch and colleagues also found that almost all patients benefited from the use of physiotherapy, highlighting the benefits of early and consistent care by physiotherapists. These interventions make a significant contribution to supporting patients through a challenging time in their lives. However, the authors emphasised the need for the further expansion of education and training for physiotherapists in palliative care [5].

As part of a systematic literature review by Kathrin Woitha et al. on the development and use of physiotherapy in palliative care, a targeted search was conducted in various databases. In addition, German and English textbooks and journals on physiotherapy and palliative care were consulted. This analysis showed that physiotherapy is increasingly recognised and used in palliative care, as evidenced by the growing number of publications on the subject. It has contributed significantly to the expansion of intervention strategies in palliative care [8]. However, to fully assess the impact of physiotherapy interventions in different palliative care settings, another study by the same authors emphasised the need for further intervention studies [9]. Similarly, the role of physiotherapists in palliative care remains poorly understood [8].

To date, there has been no nationwide survey in Germany of physiotherapists working in palliative care regarding their treatment methods, areas of specialisation and professional status within the health care system. Therefore, this survey aims to fill this gap and provide a clearer overview.

The research questions of the survey are as follows:

- In which settings of palliative care are physiotherapists active (hospice, palliative care unit and outpatient palliative care at home), and what types of treatment do they provide?
- What specific treatments, modalities and techniques do physiotherapists use with palliative care patients?
- How do physiotherapists perceive their “position” in palliative care, and do they think that physiotherapy is used appropriately for palliative patients?
- What is the need for further training of physiotherapists in the specific treatment methods for palliative care patients?
- Is a time frame of approximately 20 min per physiotherapy treatment session considered sufficient for palliative care patients, or should the treatment time per patient be extended for specific types of therapy?
- What are the reasons given by the participating physiotherapists who do not work in palliative care for not working in this field, and what potential barriers or concerns do they report?

## 2. Materials and Methods

The survey of physiotherapists working in palliative care units, hospices or specialised outpatient palliative care (German abbreviation: SAPV) in Germany was conducted using an online questionnaire via the SurveyMonkey^®^ platform (San Mateo, CA, USA). The questionnaire was developed by our research group and was based on a previously published survey on occupational therapists in palliative care. The structure and topics were adapted to the physiotherapy context, taking into account the team’s professional experience and prior research. While no formal pilot testing was conducted, the format and mode of delivery had already been successfully applied in the earlier study. The study design, content, recruitment procedures and data management are in accordance with the ICH Guidelines of Good Clinical Practice (GCP) and were reviewed and approved by the Ethics Committee of the Department of Human Medicine at the Philipps University of Marburg (No. 23/06). This study was registered in the German Clinical Trials Register (DRKS00031188). According to German law, a signed consent form is not required due to the anonymous nature of the survey. The Checklist for Reporting of Survey Studies (CROSS) was used as a reporting checklist for manuscript preparation.

### 2.1. Design of the Questionnaire

The questionnaire consisted of twenty-two questions. To minimise potential bias caused by targeting “only physiotherapists working in palliative care”, it was initially distributed to all physiotherapists.

The first four questions of the questionnaire collected basic demographic and professional information about the physiotherapists surveyed. This included their gender, age, professional qualification and the number of years working as a physiotherapist.

Following these questions, the fifth question acted as a filter, asking respondents to indicate whether they work in palliative care or not. Physiotherapists who did not work with palliative care patients were asked about the reasons for this and their interest in working in the palliative care sector.

For physiotherapists working with palliative care patients, the questionnaire continued with specific aspects of their professional activities. These included questions about their years of experience in palliative care, whether they work with children or adults and details of their current employment status. Respondents were also asked to indicate the settings in which they provide care for palliative patients (e.g., hospice, home visits, etc.).

In addition, participants were asked about their specific palliative care responsibilities, including the average duration of treatment per patient and whether they perceive a need for extended treatment times. They were also asked to describe their role in palliative care and share their preferences regarding workload in this area.

The questionnaire also explored challenging situations in their daily work and assessed whether their physiotherapy expertise is adequately utilised in palliative care settings. Finally, the participants were asked to share their personal experiences of palliative physiotherapy and their views on the need for further research, education and training initiatives in this specialised field.

### 2.2. Sampling and Recruitment

To participate in the survey, respondents had to be either in training or studying to become a physiotherapist or already a graduate and practising as a physiotherapist. The advanced section of the questionnaire was specifically designed for those who work professionally with palliative care patients.

Data collection took place between 27 January and 31 May 2023. Members of the German Association for Physiotherapy were contacted on 27 January, 3 March and 7 April 2023, via an internal weekly info mail. In addition, a call for participation was made on the official website of the professional association (www.physio-deutschland.de) on 3 March and 7 April 2023.

On 30 January 2023, the Physiotherapy, Occupational Therapy and Speech Therapy Section of the German Society for Palliative Medicine (DGP) was asked to distribute the survey to its members. On 14 April 2023, an email was sent to all hospices, specialised outpatient palliative care services and palliative care units in Germany, inviting physiotherapists working in these settings to participate in the survey.

### 2.3. Data Analysis

All returned questionnaires were first checked for completeness. Due to the configuration of the SurveyMonkey^®^ server, duplicate responses were automatically prevented. Quantitative data were analysed descriptively using frequency and percentage distributions with Microsoft Excel^®^ version 16.78. The free-text responses addressed key topics of the study and aimed to gather additional perspectives from participants. The content of these questions was closely aligned with the research questions but was presented individually in the survey. These responses were reviewed independently by the research team, recurring themes were identified, and the core content was summarised thematically by consensus. The aim was not to conduct a methodologically controlled qualitative analysis but rather to provide a structured overview of the central perspectives and experiences of the respondents, supported by illustrative original quotes.

## 3. Results

A total of 450 valid responses were received during the four-month survey period. Question five served as the primary selection question: of the respondents, 349 (77.6%) indicated that they worked in palliative care. Of the remaining 101 (22.4%) respondents not working in palliative care, 49 expressed an interest in working with palliative care patients.

**Figure 1:**
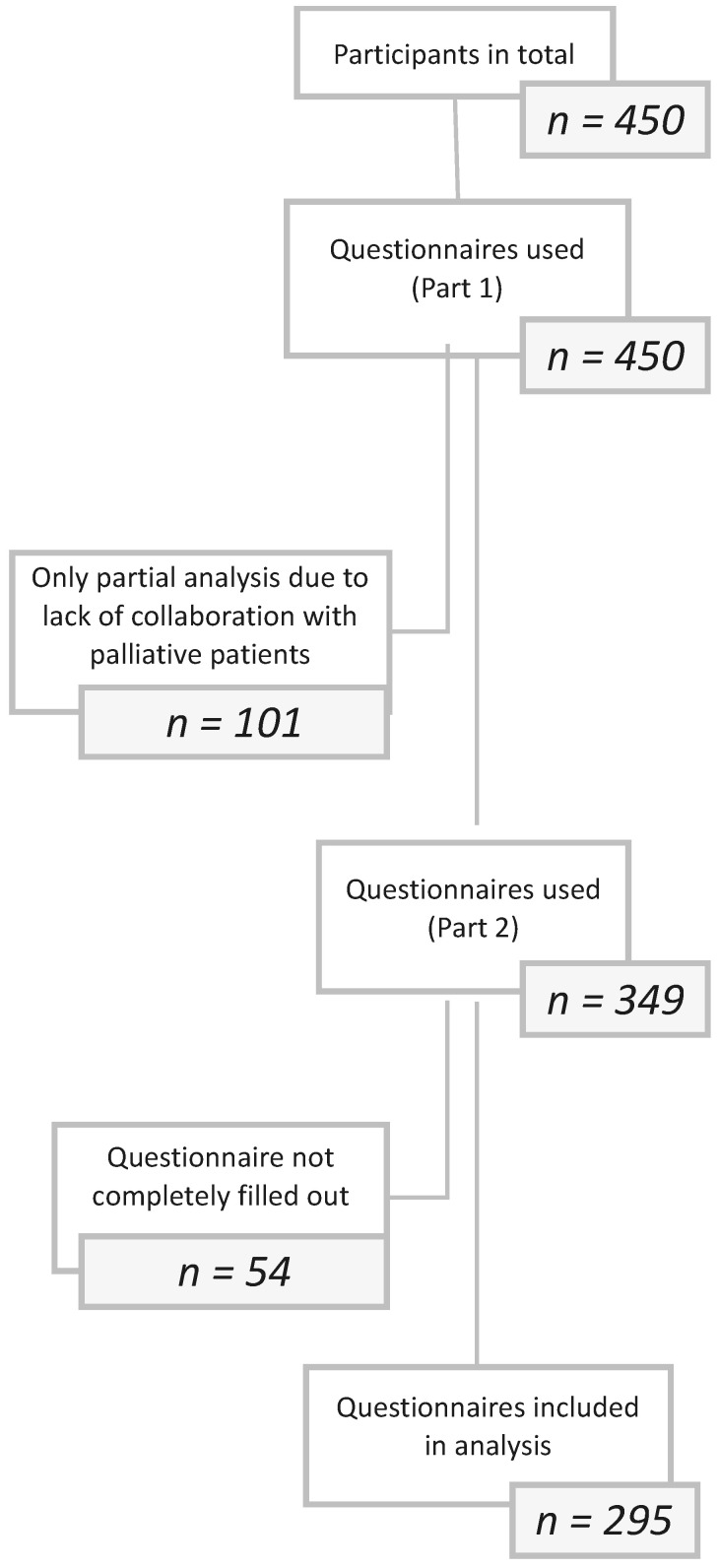
Flowchart showing the included questionnaires.

Of the 349 participants working in palliative care, 54 (15.5%) either did not complete the questionnaire in full or abandoned it. Therefore, 295 (84.5%) completed questionnaires were eligible for detailed analysis (see Figure 1).

### 3.1. Characteristics of Respondents

Table 1 provides an overview of the demographic characteristics of all participants. The majority of the participating physical therapists were female (84.9%). Regarding the age distribution, more than 68.0% of the participants were older than 45 years. A vast majority had completed professional training or a degree in physiotherapy (86.0%).

The participating physical therapists showed extensive professional experience. In particular, 40% had more than 30 years of professional experience. In addition, 26.7% had between 21 and 30 years of experience, while only 14.3% reported less than 10 years of professional experience.

### 3.2. Scope and Tasks of Physical Therapists in Palliative Care

The following section addresses the question of which settings physiotherapists are active in within palliative care and what kinds of therapeutic services they provide in these contexts. Table 2 illustrates the different areas of work of the palliative care physiotherapists who participated in the survey. Almost half of the respondents (46.8%) work in more than one area. A significant proportion of respondents (43.4%) carry out home visits outside of specialised outpatient palliative care services, while many physiotherapists (27.8%) care for palliative patients in hospitals outside of palliative care units. In contrast, fewer physiotherapists work in hospices (17.0%) or specialised outpatient palliative care services (10.2%) at home.

The majority of physical therapists work mainly with adult patients (92.9%), while a smaller percentage (10.6%) work with children and adolescents. Employment is evenly distributed among participants, with 32.5% working part-time, 33.9% working full-time, and 34.2% self-employed.

**Figure 2:**
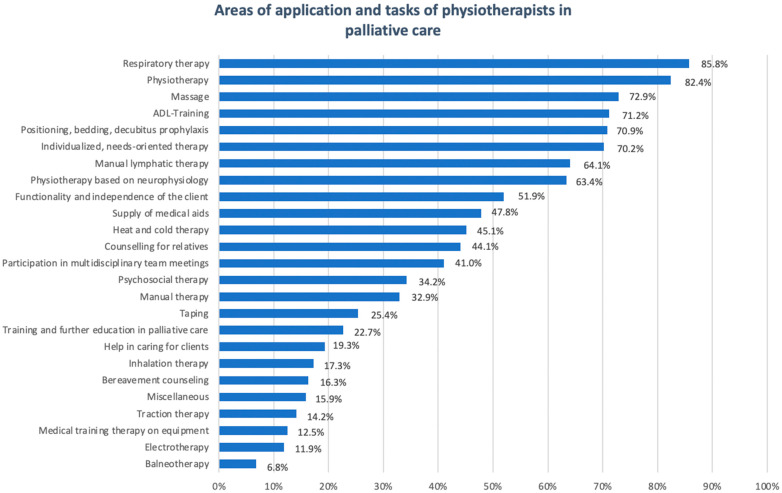
Physiotherapeutic roles in palliative care.

Figure 2 illustrates the different applications and tasks of physiotherapists in palliative care, including direct and indirect activities such as family counselling or interdisciplinary coordination. Among direct patient activities, respiratory therapy emerges as the primary task, performed by 85.8% of physiotherapists.

Adaptive therapy, which involves tailoring therapeutic interventions to the individual patient’s needs, is a central aspect of the therapeutic spectrum, involving 70.2% of respondents. Equally important is support for patients in their daily lives, including the provision of necessary aids, a service offered by almost half of respondents (47.8%).

Activities that are not directly related to patient care include counselling relatives, a task performed by 44.1% of physical therapists. Organisational tasks, such as participation in interdisciplinary teams or specific education and training tasks, play a more limited role.

More specialised forms of therapy, such as balneotherapy and electrotherapy, complete the range of treatments.

### 3.3. Free-Text Answers

For some questions, participants were given the opportunity to provide free-text responses in addition to the pre-defined response options. In particular, one question asked physiotherapists to describe in detail their understanding of their role in palliative care. These free-text responses were then categorised into five main themes using a qualitative analysis approach. Below are examples of these identified themes, together with selected extracts from the free-text responses.

#### 3.3.1. Topic I: Treatment Time

Respondents to the survey expressed a desire for more time to provide therapy, which would allow them to be flexible in responding to patients’ day-to-day needs. They emphasised the importance of a holistic approach that goes beyond physical treatment to include psychosocial aspects, such as intensive discussion and support for family members. They also highlighted the need for sufficient time to effectively implement specialised treatments such as lymphatic drainage, respiratory therapy and ADL training:

“Treating people in palliative care requires a different type of interaction and approach compared to treatment in a physiotherapy practice. Time is also an important factor.”

(N-19)

“I believe that in a palliative care setting each patient should be allocated a minimum treatment time of 60 min. There is nothing worse than therapists who are under time pressure and are unable to provide a needs-based therapy.”

(N-57)

“Depending on the prescribing doctor, physiotherapy and/or massage are often prescribed initially. However, these prescriptions can be adjusted, as patients often receive them when they are nearing the end of life. In such cases, manual lymphatic drainage and respiratory therapy (sometimes including reflexive respiratory therapy) may be more appropriate. Positioning or conversation, especially with relatives, may also be more beneficial. The time allotted is often too short, especially for providing adequate ‘support’.”

(N-252)

#### 3.3.2. Topic II: Personal Role and Work in Palliative Care

The participating physiotherapists emphasised their role as a supportive companion, not only for the patient but also for their relatives. They see themselves as important interlocutors and facilitators, promoting communication between relatives and the interdisciplinary team to ensure comprehensive and coordinated care. However, some respondents reported feeling undervalued in their role as physiotherapists, citing insufficient recognition and integration into the treatment team.

The main aim of physiotherapy treatment in palliative care is usually to improve the patient’s wellbeing and reduce pain:

“It is very valuable to give patients a little ‘better’ time in their lives through therapy (pain relief, relaxation, perhaps also time to feel good) and someone to listen, distract, or share silence with them, as the patients wish.”

(N-67)

“As a therapist who primarily focuses on the symptoms (psychological and physical) of the patients, but also acts as a bridge to the nursing staff and doctors”

(N-163)

“For me, physiotherapy in palliative care means, in addition to medical and nursing care, an accompanying, supportive, and needs-oriented application of physiotherapeutic measures and activities with patients with incurable diseases, with the aim of achieving or maintaining the best possible quality of life until the end of life.”

(N-235)

#### 3.3.3. Topic III: Stressful Situations in Everyday Life

Physiotherapists highlighted several stressful situations in their daily work, in particular the emotional challenges of caring for young patients, those who die prematurely and supporting the children they leave behind. These scenarios impose a significant emotional burden and illustrate the difficulty of maintaining appropriate emotional boundaries.

In addition, the lack of time is cited as a significant factor that exacerbates these burdens and makes it difficult to respond adequately to the needs of affected patients and their families. Taken together, these issues represent a significant burden in a daily practice and highlight the need to develop support services and strategies to address these challenges:

“When I feel powerless and can’t find words or actions that might help.”

(N-7)

“If I lack personal distance, the situation gets under my skin.”

(N-417)

“End-of-life care can often be stressful because you develop personal relationships with the patients, especially when they are of a similar age or have similar social structures (such as children) that they leave behind.”

(N-30)

“Young patients who are in the prime of their lives and may have children. It’s a burden, and it’s so difficult to find the right words to support them and to hold back your own emotions.”

(N-98)

“I’m not yet very good at leaving the grief and emotional distress ’at work’.”

(N-19)

“The lack of time for the patient and their relatives, who need a lot of care and attention.”

(N-232)

#### 3.3.4. Topic IV: Utilisation of Physiotherapy Expertise

Participants highlighted a lack of interdisciplinary exchange within the multi-professional team, which hinders the effective integration and use of physiotherapy expertise in patient-centred care. They also highlighted the limited recognition of physiotherapy work, which limits its potential contribution to palliative care:

“I still come across situations where doctors and nurses are unaware of what we physiotherapists can do and how much we contribute to palliative care. The diversity of my treatment options is rarely asked for in detail. Doctors and nurses are often surprised by the full extent of what we physiotherapists can do.”

(N-125)

“Our work is often perceived as a physical activity and the complexity of our skills is not recognised from the outside. However, the patients and some of their relatives are aware of and appreciate our wide range of services and treatment methods.”

(N-434)

“Neither society nor education has fully recognised how important we are in this setting. Even most doctors outside palliative care units hardly see the need. They often associate our profession with completely different approaches and treatments.”

(N-449)

#### 3.3.5. Topic V: Education and Training Needs

When asked about their training needs in palliative physiotherapy, respondents expressed an interest in additional psychological training, particular in dealing with issues related to death and bereavement. In addition, 63.4% emphasised the need for further research in this area, while 25.4% expressed a willingness to participate in research. Respondents highlighted the importance of developing skills that balance empathy and professionalism while safeguarding their own emotional wellbeing. They also identified a need for training in self-care, setting emotional boundaries and building resilience. Participants also expressed an interest in complementary and alternative therapies to better meet the diverse needs of their patients:

“Dealing with loss and grief when a patient dies. Timely recognition of complications in palliative patients (e.g., after chemotherapy), including the development of a palliative care flag system.”

(N-195)

“Perhaps all of this could be included in the spirit of a ‘Last Aid Course’? (…) Alternative aids and remedies, such as aromatherapy, incense, homeopathy, yoga, mudras, and mantras. (…) New topics could include: How do I deal with it? How can I bear it? How can I help without imposing? How can I recognise what is really important? How can I be a rock in the sea of life and death? What effect could it have on me, and do I even want it to?”

(N-252)

“Verbal and non-verbal communication, ethical issues, self-awareness, pain relief techniques”

(N-283)

“The dying process from a physiological and medical perspective, training in communication skills, and seminars to raise awareness of one’s own approach to the subject of death and dying, including consideration of cultural differences.”

(N-406)

### 3.4. What Non-Palliative-Care Physiotherapists Think About Palliative Care Work

Of the 101 respondents who do not work with palliative care patients, 86 responded to the question of whether they would work in this area. Of these, 59.8% were interested, but cited various barriers. These included structural, personal and training barriers. Many feel constrained by their current working environment due to a lack of job opportunities and a shortage of palliative care patients. In addition, they feel unprepared and inadequately equipped to meet the complex needs of palliative care patients due to a lack of specific training. Personal reasons, such as limited time, discomfort with death and satisfaction with their current position also contribute to their lack of involvement in palliative care.

Of those surveyed, 40.2% do not want to work with palliative care patients. The main reasons given were psychological strain and specialisation in other fields. Many feel unable to cope with the emotional demands due to personal grief or past experiences. In addition, many work in areas such as orthopaedics, traumatology or geriatrics and therefore prefer success-oriented work with visible progress, which is often lacking in palliative care. Organisational factors also play a role, such as a lack of capacity for home visits and a preference for working in physiotherapy practices, where palliative care patients are rare.

## 4. Discussion

The results of our survey suggest that, from the perspective of physiotherapists, physiotherapy plays an important role in supporting the quality of life of seriously ill patients. In particular, they described the necessity of adapting treatment to the daily condition of each patient, which includes both physical and psychosocial aspects. This perceived role is reflected in the variety of reported treatment approaches, ranging from respiratory therapy to training in activities of daily living. These findings are in line with Hanks et al., who similarly emphasised the relevance of symptom control, functional rehabilitation and psychological support by physiotherapists [10]. Compared to the study by Möller et al., which investigated the experiences of eight physiotherapists, our analysis based on 295 participating physiotherapists who work in palliative care offers a broader perspective on the role of physiotherapy in palliative care. Both studies, despite different sample sizes, confirm the importance of respiratory therapy and individualised, needs-based therapy [11].

This aligns with the responses in our survey, where participants mentioned the need for individually tailored physiotherapy to address patients’ varying needs. The importance of individualised physiotherapy support in palliative care is also highlighted by Boddenberg and Böhm. Their work shows that targeted physiotherapy intervention can lead to significant benefits for palliative care patients. They note that even small improvements in mobility can increase independence and reduce the need for care. In addition, professional physiotherapy can improve quality of life, self-control, satisfaction and social functioning and reduce anxiety [12]. Physiotherapy thus plays a crucial role in maintaining a patient’s dignity and quality of life despite physical decline [13]. Some participants described aspects of their work that extend beyond physical treatment, such as being a supportive presence and accompanying patients and their families during emotionally challenging situations.

Kaur and Bernabeu-Wittel also emphasise that physiotherapy goes beyond symptom control and contributes to the dignity of the patient [14,15]. These psychosocial aspects are essential to enable patients to live with dignity and self-determination until the end of life. Physiotherapists act as trusted companions, fostering close relationships with patients through physical proximity and providing support during this challenging time.

Recent research has highlighted the complexity of dignity-related distress in palliative care. The findings highlight that challenges such as loss of autonomy, value, meaning and physical discomfort are central to the experience of palliative care patients. This emphasises the need for holistic treatment that addresses both physical symptoms and psychosocial aspects [16].

In palliative care, physiotherapists work closely with patients and their families, balancing the patient’s wellbeing with the realities of progressive illness [17]. This adaptability is essential, as it allows therapists to provide ongoing and effective support throughout the patients’ life.

In our survey, respondents described their involvement in various interactive and supportive roles, including family counselling and interdisciplinary communication. This reflects the findings of Möller et al., who also highlighted the multiple roles of physiotherapists in specialised palliative care, particularly in family education and support [11]. These roles complement specialised therapeutic approaches and emphasise the holistic approach of palliative care, which goes beyond direct patient care to include the family support and teamwork.

Respondents also identified flexible therapy as a key factor in improving the quality of life of palliative care patients, as outlined in the *Oxford Textbook of Palliative Medicine*. Physiotherapists, through their adaptive and patient-centred approaches, are an indispensable pillar of the interdisciplinary palliative care team and make a significant contribution to preserving patients’ dignity and wellbeing [10]. In addition to physiotherapists, the team includes occupational therapists, nurses, social workers, art and music therapists, pastoral care workers, theologians, volunteers, physicians and psychologists—all contributing to holistic palliative care [18].

In our survey, several participants reported that physiotherapists are not yet fully integrated into interdisciplinary palliative care teams, and that their expertise is often underutilised. This reflects the findings of Wilson et al., who point out that, unlike the team-based approaches in inpatient rehabilitation centres or structured discharge discussions in acute care hospitals, the regular involvement of physiotherapists in interdisciplinary hospice and palliative care teams is not yet common practice [19]. Nevertheless, interdisciplinary collaboration between different disciplines remains essential for the comprehensive care of dying patients. Clear communication within the team is essential to address the complex and rapidly changing physical, emotional and social needs of patients. Recognising the equal value of all team members improves care and ensures that patients and their families receive the best possible support [20].

Early involvement of physiotherapists in palliative care, as discussed by McLeod and Norman, may allow for more effective symptom control and better tailoring of care strategies to patients’ needs. The development of support networks for physiotherapists not only enhances professional competence but also facilitates a coordinated approach within the interdisciplinary team [21].

Beyond the limited integration, some respondents in our survey also highlighted misconceptions about physiotherapy in palliative care. They reported that the profession is often narrowly associated with mobilisation or functional restoration, which contrasts sharply with the need for symptom-based and supportive interventions.

Several respondents noted that they often encounter the belief that “nothing more can be done” for palliative patients. This perception can lead to physiotherapy services being undervalued and underused. Respondents emphasised that they are often involved too late in the care process or that their expertise is not fully utilised. The systematic review by Putt et al. also concluded that physiotherapy interventions remain underused in palliative care, despite their crucial role in the interdisciplinary team [22]. This gap needs to be addressed to ensure optimal support for patients and their families and to strengthen the team as a whole.

These observations are supported by a qualitative study by Wilson et al., which shows that early and more extensive involvement of physiotherapy can improve the quality of life of palliative care patients and their families. According to the authors, establishing the early integration of physiotherapy into the care process as standard practice is necessary to realise its full potential in palliative care [23].

Path and Nieland emphasise that it is a misconception to believe that physiotherapy is reserved exclusively for patients with curative intentions. On the contrary, in palliative care, physiotherapy is an essential component in providing relief and support to patients. Although the benefits of such interventions are often temporary, their value should not be underestimated. Patients in palliative care benefit significantly from the effects of physiotherapy in helping them to cope with the challenges of a progressive illness [13].

Another critical issue that emerged from our results was the amount of time available to treat palliative care patients; 46.4% of respondents expressed a desire for more time per session. This raises important questions about current practice and billing arrangements in palliative care and suggests a need to re-evaluate the framework for physiotherapy services in this area. However, there are no current studies that specifically address the time component of physiotherapy for palliative care patients.

In the German outpatient sector, a doctor’s prescription is required for any form of therapy. Since 2019, a special prescription option for “complex physiotherapeutic treatment for palliative patients” has been available for patients with private health insurance in Germany. This allows therapists to bill for 60 min per patient, which is a significant difference from the usual 20 min per standard treatment [24,25]. These extended time slots are crucial for meeting the complex needs of palliative care patients and highlight the need for more flexible arrangements for patients with statutory health insurance, as proposed by the German Medical Association (Spitzenverband der Heilmittelverbände e.V. (SHV)). However, a federal committee rejected this proposed flexibility, despite broad support from medical associations, highlighting a gap between administrative regulations and the clinical needs of palliative care [26].

The survey responses highlight the emotional and psychological stress that physiotherapists experience when working with critically ill and terminally ill patients. A qualitative study of Australian physiotherapists supports these findings, showing that dealing with psychologically distressed patients is common and that empathic engagement can lead to significant stress. This is echoed in the open-ended responses from our survey. The concept of empathic stress, as described by McGrath, supports the idea that palliative care physiotherapists face unique emotional and psychological challenges [27].

Conversely, the personal narratives from McLeod and Norman’s study show that working with palliative care patients provides a deep sense of purpose beyond functional rehabilitation. Despite the emotional difficulties, physiotherapists report finding fulfilment in making a direct, positive impact on the quality of life of patients and their families [21]. Eckersley and Taylor’s study shows that resilience can protect against burnout, suggesting that targeted resilience training and support mechanisms can help to strengthen therapists’ psychological resilience [28]. In this context, 63.4% of the physiotherapists surveyed expressed a desire for more research and additional training in palliative physiotherapy, particularly focusing on psychological aspects and working with dying patients. They highlighted the need for training in self-protection, emotional detachment, resilience and complementary therapies to enhance their care.

A survey of occupational therapists conducted by our working group revealed similar results regarding professional recognition and the utilisation of therapeutic expertise in palliative care [29]. These parallels underline the need for greater interdisciplinary collaboration and a holistic approach to palliative care.

In line with this, Anna Pape’s study on physiotherapy and occupational therapy in German palliative care highlighted the need for further research to clarify the role of both therapies and to raise awareness of their work [25]. This emphasises the urgency of improving interdisciplinary collaboration and training to ensure patient-centred and needs-based care for palliative care patients.

### Limitations

This study was conducted as an anonymous online survey, allowing participants to skip questions if they wished. As a result, not all participants completed the full questionnaire. While the anonymity of the survey may have encouraged honest responses, skipped questions may have influenced the overall results. In addition, this study only represents the views of physiotherapists and does not include the views of other professionals or of patients themselves. Furthermore, the impact of physiotherapy on the wellbeing of palliative care patients cannot be determined from this survey alone. Further research, including controlled intervention studies, is needed to investigate these effects.

## 5. Conclusions

The results of our survey indicate that, from the perspective of the participating physiotherapists, physiotherapy plays an important role in palliative care that goes beyond physical treatment and rehabilitation—particularly through contributions to quality of life, psychosocial support and individual accompaniment, as described in the free-text responses. The provision of needs-based and flexible therapy is essential to support patients’ wellbeing through to the end of life.

From the perspective of the participating physiotherapists, there is a clear need for better training and earlier involvement in palliative care in order to strengthen their skills and fully utilise their therapeutic potential. An adequate duration of therapy, an understanding of the psychosocial aspects of care and the recognition of physiotherapy expertise within the interdisciplinary team are essential to improve the quality of palliative care.

Organisational and temporal frameworks should be reassessed and adapted to ensure appropriate care. In addition, the emotional and psychological burden on physiotherapists requires greater attention and support to sustain their work.

Finally, the survey points to the need for further research into physiotherapy in palliative care to continually improve the quality of care and increase the recognition of the role of physiotherapy in this area.

## Figures and Tables

**Table 1:** Characteristics of the respondents.

		*n* = 450	Percentage
Gender	Female	382	84.9
	Male	68	15.1
	Divers	0	0.0
Age (years)	<25	10	2.2
	25–35	56	12.4
	36–45	78	17.3
	46–55	136	30.2
	>55	170	37.8
Qualifications	Currently in training/studying	7	1.6
	Completed vocational training	387	86.0
	Bachelor’s degree	31	6.9
	Master’s degree	10	2.2
	Others	15	3.3
Years of experience	<5 years	30	6.7
	5 to 10 years	34	7.6
	11 to 20 years	82	18.2
	21 to 30 years	120	26.7
	>30 years	184	40.9
Working with palliatve clients	Yes	349	77.6
	No	101	22.4

**Table 2:** Distribution of palliative work.

		*n* = 295	Percentage
Number of years working with palliative clients	<5 years	75	25.4
	5 to 10 years	69	23.4
	11 to 20 years	84	28.5
	21 to 30 years	37	12.5
	>30 years	30	10.2
Primary collaboration	Children/adolescents	32	10.9
	Adults	274	92.9
Employment relationship	Part-time employed	96	32.5
	Full-time employed	100	33.9
	Self-employed	101	34.2
	Voluntary work	13	4.4
	Others	9	3.1
Working area	Clinic (outside a palliative care unit)	82	27.8
	Palliative care unit	93	31.5
	Hospice	50	17.0
	Specialized outpatient palliative care team	30	10.2
	Medical practice	88	29.8
	Home visit (outside specialized palliative care)	128	43.4

## Data Availability

The data are available upon reasonable request from the authors.
